# Interaction effects of sleep duration and activities of daily living on depressive symptoms among Chinese middle-aged and older adult individuals: evidence from the CHARLS

**DOI:** 10.3389/fpubh.2025.1547329

**Published:** 2025-03-12

**Authors:** Tianmeng Wang, Wenjin Han, Caihua Wang, Yanqing Kang, Yaping Wang, Shuangyan Lei, Zhaozhao Hui, Ning Li, Xiaoqin Wang

**Affiliations:** ^1^Department of Nursing, Xi’an Jiaotong University Health Science Center, Xi’an, China; ^2^Medical School, Xi’an Peihua University, Xi’an, China; ^3^Department of Radiotherapy, Shaanxi Provincial Cancer Hospital, Xi’an, China; ^4^School of Public Health, Xi’an Jiaotong University Health Science Center, Xi’an, China; ^5^The First Affiliated Hospital of Xi’an Jiaotong University, Xi’an, China

**Keywords:** depressive symptoms, sleep duration, activities of daily living, interaction, CHARLS

## Abstract

**Objectives:**

Evidence on the combined effect of sleep duration and activities of daily living (ADL) on depressive symptoms is scarce. This study aimed to explore the interaction effects between sleep duration and ADL limitations on depressive symptoms among Chinese individuals aged ≥45 years.

**Methods:**

Data were extracted from the China Health and Retirement Longitudinal Study (CHARLS) wave 2020. Sleep duration was self-reported. The Center for Epidemiological Studies Depression Scale and a 12-item scale were employed to estimate depressive symptoms and ADL limitations, respectively. Logistic regression analysis was conducted to examine the interaction effects between sleep duration and ADL limitations on depressive symptoms.

**Results:**

Logistic regression found that short sleep (OR = 1.69, 95% CI: 1.57–1.83), long sleep (OR = 0.87, 95% CI: 0.79–0.95), and ADL limitations [basic activities of daily living (BADL), OR = 1.82, 95% CI: 1.66–2.01; instrumental activities of daily living (IADL), OR = 1.88, 95% CI: 1.71–2.07] were associated with depressive symptoms. Furthermore, synergistic interaction effects on the depressive symptoms risk were identified between short sleep and IADL limitations (RERI = 1.08, 95% CI: 0.57–1.59) or BADL limitations (RERI = 1.13, 95% CI: 0.60–1.65). Conversely, antagonistic interaction effects were observed between long sleep and IADL limitations (RERI = 0.88, 95% CI: 0.39–1.38) or BADL limitations (RERI = 0.76, 95% CI: 0.25–1.27) on depressive symptoms.

**Conclusion:**

The study revealed significant interactions between sleep duration and ADL limitations on depressive symptoms, suggesting that enhancing ADL’s function and ensuring adequate sleep duration could effectively prevent depressive symptoms.

## 1 Introduction

Depression is a common mental disorder, characterized by a low mood or the loss of pleasure in activities for a long period (1). Globally, approximately 280 million individuals suffer from depression (1), with an estimated 41.01 million affected in China, ranking as the second highest number worldwide (2). It is projected that depression will be the leading global disease burden by 2030 (3). Depression can interfere with daily life (1), lead to illnesses (e.g., diabetes, cardiovascular disease, disability, etc.) (4, 5), and even increase the premature mortality risk (6). Depressive symptoms, as an early sign of depression (7), encompass a range of emotional and physical symptoms that may not meet the full criteria for diagnosing depression (8). Current research has shown that the prevalence of depressive symptoms among Chinese middle-aged and older individuals has escalated to 37.62% (9), posing a tremendous burden on families and society. Thus, early identification of risk factors for depressive symptoms is imperative for preventing and delaying depression (10). Recent systematic review (11) and meta-analysis (12) have highlighted several modifiable risk factors associated with depressive symptoms, including lifestyle behaviors (e.g., physical activity), physical health-related conditions (e.g., chronic disease and activities of daily living), and psychosocial factors (e.g., social support). Among these, activities of daily living have been recognized as a crucial factor, garnering considerable attention from scholars (11, 12).

Activities of daily living (ADL) refer to an individual’s daily self-care activities, categorized into basic activities of daily living (BADL) and instrumental activities of daily living (IADL) (13). BADL includes fundamental skills necessary for managing one’s basic physical needs (e.g., dressing, bathing, and eating); whereas IADL involves more complex tasks in the community (e.g., doing housework and cooking) (13). In recent years, accumulating longitudinal studies have examined the association of ADL with depressive symptoms, finding that more severe ADL limitations predicted a higher risk of depressive symptoms (14, 15). This suggests that ADL has become a significant predictor of depressive symptoms (16). A plausible explanation is that ADL limitations act as psychological stressors by restricting individuals’ independence in self-care (15) and social relationships (14, 15), ultimately resulting in the onset of depressive symptoms (15, 17). Furthermore, depressive symptoms can, in turn, aggravate ADL limitations (18), potentially creating a vicious circle.

Sleep is a fundamental behavior essential for physical health and cognitive function, characterized by specific brain electrical activity that undergoes predictable changes across the lifespan (19). Considerable evidence has demonstrated that sleep duration was correlated to numerous health problems, such as metabolic syndrome (20), cancer (21), and suicidal behavior (22). However, the relationship between sleep duration and depressive symptoms remains unclear, with numerous studies yielding inconsistent results (23–27). Some studies have indicated that both shorter and longer sleep increased the risk of depressive symptoms among middle-aged and older individuals, showing a U-shaped relationship (23, 24). Others have shown that only short sleep is significantly associated with depressive symptoms (25, 26). However, a 4-year longitudinal study found that long sleep could reduce the risk of depressive symptoms (27). In view of this, the relationship between long sleep and depressive symptoms warrants further exploration.

In summary, mounting studies have delved into the independent associations of sleep duration and ADL with depressive symptoms. Few studies have examined the combined effects of sleep duration and ADL limitations on depressive symptoms and their potential mechanisms. To date, one cross-sectional study identified a multiplicative interaction between sleep duration and ADL limitations on depressive symptoms (28). However, it did not address the additive interaction, which could provide a different perspective on the cumulative effects of these factors on depressive symptoms. Given this gap in the literature, the present study aimed to investigate the additive interaction effects of sleep duration and ADL limitations on depressive symptoms, potentially providing valuable insights into prevention and intervention strategies.

## 2 Materials and methods

### 2.1 Study population

This study used data from the China Health and Retirement Longitudinal Study (CHARLS). The CHARLS is a nationally representative longitudinal study targeting individuals aged ≥ 45 years in China, aimed at establishing a high-quality public database. The data collected covers various dimensions relevant to aging research, including socioeconomic status, health conditions, and other aspects. The baseline national survey was conducted in 2011, followed by regular follow-up survey in 2013, 2015, 2018, and 2020. The retention rates in each follow-up survey were above 86%. For a detailed description of CHARLS, additional information can be found on the project’s official website: http://charls.pku.edu.cn/.

The present study is based on the latest data collected in 2020, which included 19,395 participants in total. After excluding participants with missing data on basic demographic information (*n* = 692), depressive symptoms (*n* = 3,016), and sleep duration (*n* = 176), a final sample of 15,511 participants was included in the analysis.

### 2.2 Assessment of depressive symptoms

Depressive symptoms were estimated using the 10-item Center for Epidemiological Studies Depression Scale (CES-D). This tool is designed to assess a person’s mood and behavioral patterns related to depressive symptoms in the past week, which has demonstrated good reliability and validity in China (29). Each item is scored on a four-point scale ranging from 0 (rarely or none of the time) to 3 (mostly or all the time), with a total score ranging from 0 to 30 points. A higher score indicates more severe depressive symptoms. According to previous studies (30), participants with a score of 10 or higher were classified as exhibiting depressive symptoms in this study.

### 2.3 Assessment of ADL

The assessment of ADL was conducted using a 12-item scale, including six items of BADL (i.e., dressing, bathing, eating, getting into and out of bed, toileting, and controlling urination and defecation), and six items of IADL (i.e., doing housework, cooking, shopping for groceries, making phone calls, taking medicines, and managing finances). Each item offers four options: (1) no difficulty, (2) difficulty but can still complete, (3) difficulty and need help, and (4) unable to complete. Participants who had difficulty or were unable to perform one or more of the six items of BADL were classified as BADL limitations in this study. The same criteria were applied to determine IADL limitations.

### 2.4 Assessment of sleep duration

Sleep duration was assessed using a self-reported questionnaire that included the following sleep-related questions: “During the past month, how many hours of actual sleep did you get at night (average hours per night)? This may be shorter than the number of hours you spend in bed.” Based on prior studies (26, 31, 32), a daily sleep duration of 6 to 8 h per night is recommended for Chinese middle-aged and older individuals. Therefore, sleep duration was categorized into three groups: short sleep (<6 h), normal sleep (≥6 h and < 8 h), and long sleep (≥8 h).

### 2.5 Covariates assessment

The covariates encompassed demographic data, such as age (45–59 years, ≥60 years), gender (male, female), marital status (married, others including divorced, widowed, separated, and never married), residence (urban, rural), education level (primary school or below, secondary school, senior high school or above). Additionally, behavioral factors were collected, including smoking status (never smoked, currently smoking, former smoker), alcohol consumption frequency (>1 time per month, <1 time per month, no consumption), and the number of chronic diseases reported (0, 1, 2, and ≥ 3). The current study controlled potential confounders in the analysis. Model 1 was a crude model. Model 2 adjusted for age, gender, residence, marital status, education level, smoking history, alcohol consumption, and number of chronic diseases.

### 2.6 Statistical analysis

Statistical analyses were conducted using SPSS 25.0 software, with a significant level of *p* < 0.05. Categorical variables were represented by frequencies and percentages. The baseline characteristics of participants were assessed using χ^2^ tests, categorized by the presence of depressive symptoms. To evaluate the associations of sleep duration, ADL limitations, and depressive symptoms, binary logistic regression was performed. Additionally, both multiplicative and additive interactions between sleep duration (short and long sleep) and ADL limitations (including IADL and BADL limitations) were examined in relation to depressive symptoms. Multiplicative interactions were quantified through the ratio of odds ratios (ORs) for the product terms, while additive interactions were assessed using the Excel calculation table, compiled by Andersson et al. (33), which calculated the relative excess risk due to interaction (RERI), the attributable proportion due to interaction (AP), the synergy index (S), ORs, and their corresponding 95% confidence intervals (95% CIs). An additive interaction was indicated when 95% CIs of RERI and AP excluded 0, and 95% CI of S excluded 1. Specifically, a synergistic interaction was suggested when RERI >0, AP > 0, and S > 1. Conversely, an antagonistic interaction was indicated when RERI <0, AP < 0, and S < 1. Subgroup analyses were performed based on age and gender. Finally, sensitivity analysis was performed regarding “long sleep” (≥ 9 h), defined in other literature (34, 35), to ensure the robustness of the results.

## 3 Results

### 3.1 Characteristics of all participants

As shown in Table 1, a total of 15,511 participants were included in this study, of whom 7,017 (45.2%) experienced depressive symptoms, 5,474 (35.3%) reported short sleep, and 3,364 (21.7%) reported long sleep. Additionally, 3,122 (20.1%) had BADL limitations, and 3,196 (20.6%) had IADL limitations. Chi-square tests revealed that compared with participants without depressive symptoms, those with depressive symptoms exhibited significant differences in terms of gender, residence, marital status, education level, smoking status, alcohol consumption frequency, sleep duration, BADL limitations, IADL limitations, and number of chronic diseases (all *p* < 0.001).

**Table 1:** Characteristics of study participants divided by depressive symptoms (*n*, %).

Variables	Total (*n* = 15,511)	Yes (*n* = 7,017)	No (*n* = 8,494)	χ^2^	*p*
Age group				3.72	0.054
45–59	6,705 (43.23)	2,974 (42.38)	3,731 (43.93)		
≥ 60	8,806 (56.77)	4,043 (57.62)	4,763 (56.07)		
Gender				388.75	<0.001
Male	7,473 (48.18)	2,770 (39.48)	4,703 (55.37)		
Female	8,038 (51.82)	4,247 (60.52)	3,791 (44.63)		
Residence				52.43	<0.001
Urban	5,632 (36.31)	2,332 (33.23)	3,300 (38.85)		
Rural	9,879 (63.69)	4,685 (66.77)	5,194 (61.15)		
Marital status				64.40	<0.001
Married	13,395 (86.36)	5,889 (83.92)	7,506 (88.37)		
Others	2,116 (13.64)	1,128 (16.08)	988 (11.63)		
Education level				106.41	<0.001
Primary school or below	9,691 (62.48)	4,671 (66.57)	5,020 (59.10)		
Secondary school	3,722 (24.00)	1,571 (22.39)	2,151 (25.32)		
Senior high school or above	2,098 (13.53)	775 (11.04)	1,323 (15.58)		
Smoking history				155.35	<0.001
Never smoker	9,345 (60.25)	4,603 (65.60)	4,742 (55.83)		
Currently smoker	4,098 (26.42)	1,577 (22.47)	2,521 (29.68)		
Former smoker	2,068 (13.33)	837 (11.93)	1,231 (14.49)		
Alcohol consumption				134.94	<0.001
> 1 time/month,	4,324 (27.88)	1,638 (23.34)	2,686 (31.62)		
< 1 time/month	1,539 (9.92)	704 (10.03)	835 (9.83)		
No consumption	9,648 (62.20)	4,675 (66.62)	4,973 (58.55)		
Sleep duration				552.25	<0.001
Short sleep	5,474 (35.29)	3,165 (45.10)	2,309 (27.18)		
Normal sleep	6,673 (43.02)	2,642 (37.65)	4,031 (47.46)		
Long sleep	3,364 (21.69)	1,210 (17.24)	2,154 (25.36)		
BADL^a^ limitations				715.09	<0.001
No	12,389 (79.87)	4,940 (70.40)	7,449 (87.70)		
Yes	3,122 (20.13)	2,077 (29.60)	1,045 (12.30)		
IADL^b^ limitations				720.87	<0.001
No	12,315 (79.40)	4,898 (69.80)	7,417 (87.32)		
Yes	3,196 (20.60)	2,119 (30.20)	1,077 (12.68)		
Number of chronic diseases				511.15	<0.001
0	3,020 (19.47)	984 (14.02)	2,036 (23.97)		
1	3,416 (22.02)	1,320 (18.81)	2,096 (24.68)		
2	3,029 (19.53)	1,359 (19.37)	1,670 (19.66)		
≥ 3	6,046 (38.98)	3,354 (47.80)	2,692 (31.69)		

a BADL: basic activities of daily living.

b IADL: instrumental activities of daily living.

### 3.2 Independent associations of sleep duration and ADL limitations with depressive symptoms

Table 2 showed that in crude Model 1, compared with participants with normal sleep, short sleep (OR = 2.09, 95% CI: 1.94–2.25) was significantly associated with an increased risk of depressive symptoms, whereas long sleep (OR = 0.86, 95% CI: 0.79–0.93) linked to a decreased risk of depressive symptoms. BADL limitations (OR = 3.00, 95% CI: 2.76–3.26) and IADL limitations (OR = 2.98, 95% CI: 2.75–3.23) both increased the risk of depressive symptoms. In Model 2, adjusting for age, gender residence, marital status, education level, smoking status, alcohol consumption, and number of chronic diseases, the associations between short sleep (OR = 1.78, 95% CI: 1.65–1.92), long sleep (OR = 0.87, 95% CI: 0.79–0.95), BADL limitations (OR = 2.03, 95% CI: 1.85–2.23), and IADL limitations (OR = 2.05, 95% CI: 1.87–2.25) remained significant with depressive symptoms.

**Table 2:** Binary logistic regression of ADL limitations and sleep duration for participants with depressive symptoms.

Variables	Model 1		Model 2	
	OR (95%CI)	*p*	OR (95%CI)	*p*
Sleep duration				
6–8 h	Ref		Ref	
< 6 h	2.09 (1.94–2.25)	<0.001	1.69 (1.57–1.83)	<0.001
≥ 8 h	0.86 (0.79–0.93)	<0.001	0.87 (0.79–0.95)	0.002
BADL^a^ limitations				
No	Ref		Ref	
Yes	3.00 (2.76–3.26)	<0.001	1.82 (1.66–2.01)	<0.001
IADL^b^ limitations				
No	Ref		Ref	
Yes	2.98 (2.75–3.23)	<0.001	1.88 (1.71–2.07)	<0.001

a BADL, basic activities of daily living.

b IADL, instrumental activities of daily living.

Model 1: crude.

Model 2: adjust for age, gender, residence, marital status, education level, smoking history, alcohol consumption, and number of chronic diseases.

### 3.3 The interaction effects between sleep duration and ADL limitations on depressive symptoms

#### 3.3.1 The interaction effects between short sleep and IADL or BADL limitations on depressive symptoms

Compared with the reference group (non-short sleep and independent IADL), participants with both short sleep and IADL limitations had a significantly higher risk of depressive symptoms (adjusted OR 4.24, 95% CI: 3.73–4.81), as shown in Table 3. Additive interaction analysis revealed a statistically significant positive interaction between short sleep and IADL limitations on depressive symptoms in the total population, with a RERI of 1.08 (95% CI, 0.57–1.59), an AP of 0.25 (95% CI, 0.16–0.35), and a S of 1.50 (95% CI, 1.25–1.80), indicating that the combined effect of short sleep and IADL limitations on depressive symptoms was greater than their individual effect. Similarly, a significant additive interaction between short sleep and BADL limitations was observed (adjusted RERI = 1.13, 95% CI: 0.60–1.65; AP = 0.27, 95% CI: 0.17–0.37; S = 1.54, 95% CI: 1.27–1.87). In age and gender subgroup analyses, the synergistic interaction effects were consistent with the overall population (Supplementary Tables S1, S2). However, no multiplicative interactions were observed between short sleep and IADL or BADL limitations on depressive symptoms.

**Table 3:** The interaction between short sleep and ADL limitations on depressive symptoms.

Variables	Model 1			Model 2		
	OR (95% CI)			OR (95% CI)		
Short sleep – IADL –	Ref			Ref		
Short sleep + IADL –	1.98	1.83	2.14	1.83	1.69	1.98
Short sleep – IADL +	2.69	2.41	3.00	2.33	2.08	2.62
Short sleep + IADL +	5.26	4.67	5.93	4.24	3.73	4.81
RERI	1.60	0.97	2.22	1.08	0.57	1.59
AP	0.30	0.21	0.40	0.25	0.16	0.35
S	1.60	1.35	1.89	1.50	1.25	1.80
Short sleep * IADL	0.99	0.84	1.17	0.99	0.84	1.18
Short sleep – BADL –	Ref			Ref		
Short sleep – BADL +	2.15	2.66	2.38	1.82	1.68	1.97
Short sleep + BADL –	2.65	1.96	1.82	2.27	2.02	2.55
Short sleep + BADL +	5.72	5.35	4.74	4.21	3.71	4.78
RERI	1.73	1.10	2.37	1.13	0.60	1.65
AP	0.32	0.24	0.41	0.27	0.17	0.37
S	1.66	1.40	1.97	1.54	1.27	1.87
Short sleep * BADL	1.02	0.86	1.21	1.02	0.86	1.22

BADL, basic activities of daily living; IADL, instrumental activities of daily living.

Model 1: crude.

Model 2: adjust for age, gender, residence, marital status, education level, smoking history, alcohol consumption, and number of chronic diseases.

#### 3.3.2 The interaction effects between long sleep and IADL or BADL limitations on depressive symptoms

In Table 4, this study used participants with both long sleep and independent IADL as the reference group, which had the lowest risk of depressive symptoms due to the protective effect of long sleep, compared to the other three combinations. Compared with the reference group, participants with both IADL limitations and lack of long sleep had a 3.66 times higher risk on depressive symptoms (adjusted OR 3.66, 95% CI: 3.24–4.12). The additive interaction between IADL limitations and long sleep was significant (adjusted RERI = 0.88, 95% CI: 0.39–1.38; AP = 0.24, 95% CI: 0.12–0.37; S = 1.50, 95% CI: 1.16–1.93). Similarly, compared with participants with independent BADL and long sleep, those with BADL limitations and lack of sleep had 3.52 times higher risk (adjusted OR = 3.52, 95% CI 3.13–3.97), with RERI = 0.76 (95% CI: 0.25–1.27), AP = 0.22 (95% CI: 0.08–0.35), S = 1.43 (95% CI, 1.10–1.80), indicating an antagonistic effect between long sleep and BADL limitations on depressive symptoms. The sensitivity analysis further supported the above results (Supplementary Table S5). Notably, in the subgroup analysis regarding age and gender, the additive interaction effects were significant in the population aged ≥60 years and men (Supplementary Tables S3, S4).

**Table 4:** The interaction between long sleep and ADL limitations on depressive symptoms.

Variables	Model 1			Model 2		
	OR (95% CI)			OR (95% CI)		
Long sleep + IADL –	Ref			Ref		
Long sleep – IADL –	1.54	1.41	1.69	1.47	1.34	1.61
Long sleep + IADL +	2.63	2.19	3.16	2.30	1.91	2.77
Long sleep – IADL +	4.68	4.17	5.25	3.66	3.24	4.12
RERI	1.50	0.83	2.18	0.88	0.39	1.38
AP	0.32	0.19	0.45	0.24	0.12	0.37
S	1.69	1.29	2.21	1.50	1.16	1.93
Long sleep * IADL	0.87	0.71	1.07	0.93	0.75	1.14
Long sleep + BADL –	Ref			Ref		
Long sleep – BADL –	1.52	1.39	1.66	1.46	1.33	1.60
Long sleep + BADL +	2.70	2.22	3.27	2.31	1.89	2.81
Long sleep – BADL +	4.55	4.06	5.09	3.52	3.13	3.97
RERI	1.33	0.78	1.88	0.76	0.25	1.27
AP	0.29	0.18	0.41	0.22	0.08	0.35
S	1.60	1.27	2.02	1.43	1.10	1.87
Long sleep * BADL	0.90	0.73	1.12	0.95	0.77	1.19

IADL, instrumental activities of daily living; BADL, basic activities of daily living.

Model 1: unadjusted.

Model 2: adjust for age, gender, residence, marital status, education level, smoking history, alcohol consumption, and number of chronic diseases.

## 4 Discussion

To the best of our knowledge, this is the first study exploring the potential additive interaction effects between sleep duration (categorized as short and long sleep) and ADL limitations (BADL and IADL limitations) on depressive symptoms utilizing data from the CHARLS. This study demonstrated that in the overall population, there were synergistic interaction effects between short sleep and IADL or BADL limitations on the depressive symptoms risk. In contrast, antagonistic interaction effects were identified between long sleep and IADL or BADL limitations on the depressive symptoms risk.

The present study found the additive interaction effects between sleep duration and ADL limitations on depressive symptoms. Similarly, a hospital-based cross-sectional study investigated the relationship between frailty and depressive symptoms, revealing a multiplicative interaction between sleep duration and ADL on depressive symptoms (28). However, unlike multiplicative interactions that primarily inform biological mechanisms, the additive interactions explored in the present study offer practice value for public health decision-making, particularly in guiding the prioritization of prevention strategies and resource allocation to alleviate the burden of depressive symptoms (36). Compared to the hospital-based study, which was restricted to a single hospital in China and included a relatively small sample size (*n* = 1,574), the present study utilized a nationally representative sample (*n* = 15,511), thereby strengthening the generalizability of its findings. Additionally, two studies focused on the mediating relationships among these three factors (37, 38). One 7-year prospective study demonstrated that depression partially mediated the relationship between sleep duration and IADL disability, accounting for 64.8% of the total effect of short nighttime sleep on IADL disability (38). Another cross-sectional study revealed that ADL had a mediating effect on the association between insomnia and depressive symptoms in older adults aged 65 and above (37). While these studies primarily focused on causal pathways, the present study highlighted the combined effect of sleep duration and ADL limitations on depressive symptoms, providing a new perspective on how these factors influence depressive symptoms.

It is worth emphasizing that short sleep and ADL limitations exhibited a positive additive interaction effect on the depressive symptoms risk in middle-aged and older individuals. The interpretation of these findings likely involves the following two reasons. On the one hand, short sleep itself is one of the diagnostic criteria for depression (8). It may lead to ADL limitations by impairing cognitive function (39) and increasing the risk of sarcopenia (40, 41). Meanwhile, ADL limitations may provoke depressive symptoms, perhaps because physical limitations not only hinder participation in social activities (42), thereby reducing social support (43), but also cause the loss of independence and increased reliance on others (15), both of which amplify negative emotions (e.g., shame, guilt, and depressive symptoms) (14, 18, 44). On the other hand, these interactions may be explained by underlying biological mechanisms. Firstly, short sleep can elevate pro-inflammatory cytokines (e.g., C-reactive protein and interleukin-6) by disrupting circadian rhythms and activating the hypothalamic–pituitary–adrenal (HPA) axis (45, 46). Meanwhile, ADL limitations may exacerbate chronic low-grade inflammation due to reduced physical activity (47) and the presence of concomitant chronic diseases (48). These inflammatory factors can impair the function of neurons and glial cells in brain regions involved in emotional processing, thus increasing the risk of depressive symptoms (49). Secondly, short sleep and chronic stress linked to ADL limitations stimulate HPA axis hyperactivity, resulting in elevated cortisol levels (45, 50), which are not only directly related to somatic depressive symptoms (51), but also further worsen ADL impairment (52). Finally, depressive symptoms could shorten sleep duration (53), thereby triggering a vicious cycle.

Notably, the present study found that long sleep and ADL limitations exhibited antagonistic interactions on depressive symptoms in the total population, somewhat aligning with previous evidence that nocturnal sleep of 8 to 10 h can mitigate ADL-related depressive symptoms risks (28). In other words, long sleep may weaken the association between ADL limitations and depressive symptoms. Additionally, in the age subgroup analysis, antagonistic interaction effects were observed in individuals aged 60 and older. Emerging evidence reported that 8 to 10 h of sleep can reduce the risk of ADL limitations in individuals over 90 years (54), which is associated with a decreased risk of depressive symptoms (18). This may be explained by age-related declines in melatonin levels, leading to poor sleep quality (55) and decreased sleep duration (56). Hence, longer sleep duration is necessary to facilitate energy sustainment and functional recovery (28, 54), thereby reducing the risk of depressive symptoms (15). In the gender-stratified subgroup analysis, the antagonistic interaction was observed between long sleep and BADL limitations on depressive symptoms only in men. Sex-specific inflammatory responses to long sleep may underlie this disparity. A longitudinal study demonstrated that long sleep correlates with elevated C-reactive protein (CRP) levels in adult women but not men (57), and heightened CRP can increase the risk of depressive symptoms (e.g., decreased energy and feelings of worthlessness) (58). Moreover, consistent with prior evidence (59), women (60.5%) were more likely to experience depressive symptoms than men (39.5%) in the present study, which may be attributed to gender differences in stress-coping strategies (60), roles and responsibilities (e.g., childcare and caregiving) (59), and susceptibility to depression-related genes (61). However, the underlying mechanisms regarding the relationships among long sleep, ADL limitations, and depressive symptoms remain unclear and warrant further exploration in future studies.

The present study presents several notable strengths. First, this study is the first to assess the additive interaction effects between sleep duration and ADL limitations on the depressive symptoms risk, thereby providing a new perspective for prioritizing resource allocation in prevention efforts. Second, this study is based on data from a national survey, ensuring that results are representative of Chinese middle-aged and older adult individuals. However, several limitations should be acknowledged. The data relied on self-reports, which may introduce measurement errors and misclassification. Additionally, the cross-sectional design limits causal relationships between sleep duration, ADL limitations, and depressive symptoms.

## 5 Conclusion

The present study highlights the interaction effects of sleep duration and ADL limitations on depressive symptoms among middle-aged and older individuals. If confirmed in future studies, these results will assist in providing valuable insights into prevention and intervention strategies for depression.

## Data Availability

Publicly available datasets were analyzed in this study. This data can be found here: http://charls.pku.edu.cn/.
