# Supplementary material for: Interaction effects of sleep duration and activities of daily living on depressive symptoms among Chinese middle-aged and older adult individuals: evidence from the CHARLS
Source: Front Public Health. 2025 Mar 12;13:1547329. doi: 10.3389/fpubh.2025.1547329 (PMC11949275; doi:10.3389/fpubh.2025.1547329)
Supplement: Supplementary file 1 [file Supplementary_file_1.docx]

Supplementary Material

Table S1 The interaction between short sleep and ADL limitations on depressive symptoms divided by gender.

| Variables | Men | | | Women | | |
| --- | --- | --- | --- | --- | --- | --- |
|  | OR (95% CI) | | | OR (95% CI) | | |
| Short sleep – IADL – | Ref | | | Ref | | |
| Short sleep + IADL – | 1.71 | 1.52 | 1.92 | 1.96 | 1.76 | 2.19 |
| Short sleep – IADL + | 2.31 | 1.94 | 2.75 | 2.36 | 2.02 | 2.75 |
| Short sleep + IADL + | 4.29 | 3.51 | 5.24 | 4.31 | 3.67 | 5.08 |
| RERI | 1.27 | 0.37 | 2.17 | 1.00 | 0.30 | 1.69 |
| AP | 0.30 | 0.13 | 0.46 | 0.23 | 0.10 | 0.37 |
| S | 1.63 | 1.19 | 2.23 | 1.43 | 1.13 | 1.81 |
| Short sleep * IADL | 1.09 | 0.83 | 1.42 | 0.93 | 0.75 | 1.17 |
| Short sleep – BADL – | Ref | | | Ref | | |
| Short sleep – BADL + | 1.71 | 1.52 | 1.92 | 1.93 | 1.73 | 2.15 |
| Short sleep + BADL – | 2.13 | 1.79 | 2.53 | 2.39 | 2.04 | 2.79 |
| Short sleep + BADL + | 4.08 | 3.35 | 4.99 | 4.40 | 3.73 | 5.18 |
| RERI | 1.25 | 0.40 | 2.11 | 1.08 | 0.30 | 1.86 |
| AP | 0.31 | 0.15 | 0.47 | 0.25 | 0.10 | 0.39 |
| S | 1.68 | 1.21 | 2.34 | 1.47 | 1.13 | 1.91 |
| Short sleep * BADL | 1.13 | 0.86 | 1.47 | 0.96 | 0.76 | 1.20 |

IADL, instrumental activities of daily living; BADL, basic activities of daily living.

Model: adjust for age, gender, residence, marital status, education level, smoking history, alcohol consumption, and number of chronic diseases.

Table S2 The interaction between short sleep and ADL limitations on depressive symptoms divided by age.

| Variables | 45~59 years | | | ≥60 years | | |
| --- | --- | --- | --- | --- | --- | --- |
|  | OR (95% CI) | | | OR (95% CI) | | |
| Short sleep – IADL – | Ref | | | Ref | | |
| Short sleep + IADL – | 1.80 | 1.60 | 2.03 | 1.82 | 1.64 | 2.03 |
| Short sleep – IADL + | 2.64 | 2.13 | 3.26 | 2.19 | 1.91 | 2.52 |
| Short sleep + IADL + | 5.35 | 4.08 | 7.01 | 3.97 | 3.43 | 4.60 |
| RERI | 1.91 | 0.39 | 3.43 | 0.96 | 0.32 | 1.59 |
| AP | 0.36 | 0.16 | 0.56 | 0.24 | 0.11 | 0.37 |
| S | 1.78 | 1.19 | 2.66 | 1.47 | 1.15 | 1.88 |
| Short sleep * IADL | 1.13 | 0.80 | 1.59 | 0.99 | 0.81 | 1.22 |
| Short sleep – BADL – | Ref | | | Ref | | |
| Short sleep – BADL + | 1.81 | 1.60 | 2.04 | 1.81 | 1.62 | 2.01 |
| Short sleep + BADL – | 2.49 | 2.02 | 3.07 | 2.15 | 1.87 | 2.48 |
| Short sleep + BADL + | 4.66 | 3.62 | 5.99 | 4.05 | 3.49 | 4.69 |
| RERI | 1.37 | 0.10 | 2.64 | 1.09 | 0.45 | 1.73 |
| AP | 0.29 | 0.09 | 0.50 | 0.27 | 0.14 | 0.40 |
| S | 1.60 | 1.08 | 2.36 | 1.56 | 1.22 | 1.99 |
| Short sleep * BADL | 1.04 | 0.74 | 1.45 | 1.04 | 0.85 | 1.28 |

IADL, instrumental activities of daily living; BADL, basic activities of daily living. Model: adjust for age, gender, residence, marital status, education level, smoking history, alcohol consumption, and number of chronic diseases.

Table S3 The interaction between long sleep and ADL limitations on depressive symptoms divided by gender.

| Variables | Men | | | Women | | |
| --- | --- | --- | --- | --- | --- | --- |
|  | OR (95% CI) | | | OR (95% CI) | | |
| Long sleep + IADL – | Ref | | | Ref | | |
| Long sleep – IADL – | 1.35 | 1.19 | 1.54 | 1.61 | 1.41 | 1.83 |
| Long sleep + IADL + | 2.24 | 1.72 | 2.93 | 2.35 | 1.81 | 3.06 |
| Long sleep – IADL + | 3.55 | 2.96 | 4.25 | 3.86 | 3.28 | 4.55 |
| RERI | 0.95 | 0.24 | 1.65 | 0.90 | 0.21 | 1.59 |
| AP | 0.27 | 0.09 | 0.45 | 0.23 | 0.07 | 0.40 |
| S | 1.59 | 1.08 | 2.36 | 1.46 | 1.05 | 2.03 |
| Long sleep * IADL | 0.86 | 0.63 | 1.16 | 0.98 | 0.73 | 1.30 |
| Long sleep + BADL – | Ref | | | Ref | | |
| Long sleep – BADL – | 1.35 | 1.19 | 1.53 | 1.58 | 1.39 | 1.79 |
| Long sleep + BADL + | 2.10 | 1.60 | 2.77 | 2.51 | 1.88 | 3.34 |
| Long sleep – BADL + | 3.26 | 2.73 | 3.89 | 3.82 | 3.25 | 4.49 |
| RERI | 0.81 | 0.14 | 1.47 | 0.74 | -0.04 | 1.51 |
| AP | 0.25 | 0.06 | 0.44 | 0.19 | 0.00 | 0.39 |
| S | 1.56 | 1.02 | 2.36 | 1.35 | 0.95 | 1.92 |
| Long sleep * BADL | 1.35 | 1.19 | 1.53 | 1.04 | 0.76 | 1.41 |

IADL, instrumental activities of daily living; BADL, basic activities of daily living.

Model: adjust for age, gender, residence, marital status, education level, smoking history, alcohol consumption, and number of chronic diseases.

Table S4 The interaction between long sleep and ADL limitations on depressive symptoms divided by age.

| Variables | 45~59 years | | | ≥ 60 years | | |
| --- | --- | --- | --- | --- | --- | --- |
|  | OR (95% CI) | | | OR (95% CI) | | |
| Long sleep + IADL – | Ref | | | Ref | | |
| Long sleep – IADL – | 1.45 | 1.27 | 1.66 | 1.48 | 1.31 | 1.68 |
| Long sleep + IADL + | 2.31 | 1.60 | 3.32 | 2.28 | 1.82 | 2.85 |
| Long sleep – IADL + | 4.47 | 3.60 | 5.55 | 3.43 | 2.95 | 3.98 |
| RERI | 1.71 | 0.60 | 2.82 | 0.67 | 0.08 | 1.26 |
| AP | 0.38 | 0.17 | 0.59 | 0.20 | 0.03 | 0.36 |
| S | 1.97 | 1.18 | 3.29 | 1.38 | 1.01 | 1.88 |
| Long sleep * IADL | 0.75 | 0.50 | 1.13 | 0.98 | 0.77 | 1.26 |
| Long sleep + BADL – | Ref | | | Ref | | |
| Long sleep – BADL – | 1.47 | 1.29 | 1.68 | 1.45 | 1.28 | 1.64 |
| Long sleep + BADL + | 2.54 | 1.73 | 3.73 | 2.20 | 1.74 | 2.79 |
| Long sleep – BADL + | 3.94 | 3.20 | 4.85 | 3.34 | 2.88 | 3.87 |
| RERI | 0.93 | -0.20 | 2.06 | 0.69 | 0.10 | 1.27 |
| AP | 0.24 | -0.03 | 0.50 | 0.21 | 0.04 | 0.37 |
| S | 1.46 | 0.87 | 2.45 | 1.42 | 1.02 | 1.97 |
| Long sleep * BADL | 0.95 | 0.62 | 1.45 | 0.96 | 0.74 | 1.24 |

IADL, instrumental activities of daily living; BADL, basic activities of daily living.

Model: adjust for age, gender, residence, marital status, education level, smoking history, alcohol consumption, and number of chronic diseases.

**Table S5 Sensitivity analysis for the interaction between long sleep (≥ 9h) and ADL limitations on depressive symptoms.**

| Variables | Model 1 | | | Model 2 | | |
| --- | --- | --- | --- | --- | --- | --- |
|  | OR (95% CI) | | | OR (95% CI) | | |
| Long sleep + IADL – | Ref | | | Ref | | |
| Long sleep – IADL – | 1.23 | 1.06 | 1.44 | 1.22 | 1.04 | 1.44 |
| Long sleep + IADL + | 1.84 | 1.35 | 2.51 | 1.69 | 1.23 | 2.31 |
| Long sleep – IADL + | 3.82 | 3.23 | 4.52 | 3.11 | 2.61 | 3.70 |
| RERI | 1.74 | 1.18 | 2.31 | 1.20 | 0.65 | 1.75 |
| AP | 0.46 | 0.32 | 0.60 | 0.39 | 0.22 | 0.55 |
| S | 2.62 | 1.55 | 4.43 | 2.32 | 1.28 | 4.21 |
| Long sleep * IADL | 0.59 | 0.43 | 0.82 | 0.64 | 0.46 | 0.88 |
| Long sleep + BADL – | Ref | | | Ref | | |
| Long sleep – BADL – | 1.31 | 1.12 | 1.52 | 1.30 | 1.11 | 1.52 |
| Long sleep + BADL + | 2.50 | 1.81 | 3.45 | 2.17 | 1.56 | 3.02 |
| Long sleep – BADL + | 3.97 | 3.36 | 4.70 | 3.19 | 2.68 | 3.80 |
| RERI | 1.17 | 0.41 | 1.92 | 0.72 | 0.14 | 1.31 |
| AP | 0.29 | 0.11 | 0.48 | 0.23 | 0.05 | 0.41 |
| S | 1.65 | 1.08 | 2.50 | 1.50 | 1.01 | 2.22 |
| Long sleep * BADL | 1.22 | 0.87 | 1.70 | 0.88 | 0.63 | 1.23 |

IADL, instrumental activities of daily living; BADL, basic activities of daily living.

Model 1: unadjusted. Model 2: adjust for age, gender, residence, marital status, education level, smoking history, alcohol consumption, and number of chronic diseases.
